# The Role of Social Media in Online Weight Management: Systematic Review

**DOI:** 10.2196/jmir.2852

**Published:** 2013-11-28

**Authors:** Tammy Chang, Vineet Chopra, Catherine Zhang, Susan J Woolford

**Affiliations:** ^1^University of MichiganAnn Arbor, MIUnited States

**Keywords:** Internet, systematic review, overweight, obesity, social media, weight loss

## Abstract

**Background:**

Social media applications are promising adjuncts to online weight management interventions through facilitating education, engagement, and peer support. However, the precise impact of social media on weight management is unclear.

**Objective:**

The objective of this study was to systematically describe the use and impact of social media in online weight management interventions.

**Methods:**

PubMed, PsycINFO, EMBASE, Web of Science, and Scopus were searched for English-language studies published through March 25, 2013. Additional studies were identified by searching bibliographies of electronically retrieved articles. Randomized controlled trials of online weight management interventions that included a social media component for individuals of all ages were selected. Studies were evaluated using 2 systematic scales to assess risk of bias and study quality.

**Results:**

Of 517 citations identified, 20 studies met eligibility criteria. All study participants were adults. Because the included studies varied greatly in study design and reported outcomes, meta-analysis of interventions was not attempted. Although message boards and chat rooms were the most common social media component included, their effect on weight outcomes was not reported in most studies. Only one study measured the isolated effect of social media. It found greater engagement of participants, but no difference in weight-related outcomes. In all, 65% of studies were of high quality; 15% of studies were at low risk of bias.

**Conclusions:**

Despite the widespread use of social media, few studies have quantified the effect of social media in online weight management interventions; thus, its impact is still unknown. Although social media may play a role in retaining and engaging participants, studies that are designed to measure its effect are needed to understand whether and how social media may meaningfully improve weight management.

## Introduction

Obesity is a major US public health problem that is associated with lower quality of life, stigma, medical complications, and higher health care costs [1-6]. Despite a decade of public awareness and attention, the prevalence of obesity continues to rise in some groups, a trend that reflects the complex nature of this disease and the diverse medical, social, and behavioral domains that underlie its management [7].

Over one-half of adults in the United States use social media platforms, such as Facebook, Twitter, MySpace, and LinkedIn [8]. The social support and feelings of interconnectedness individuals experience with social media help explain the prolific growth of these platforms in everyday life [9,10]. These domains are also relevant to the success of online weight-management interventions. Social media may represent a promising resource in combating obesity at a population level. Several properties of social media make it ideal for such purposes: (1) social media facilitates asynchronous communication and provides 24/7 access to participants; (2) it overcomes barriers such as transportation and distance, allowing those with mobility, speech, or hearing problems to interact in online interventions; and (3) given the relative anonymity to discuss sensitive topics, social media is ideally suited for stigmatizing conditions such as obesity. However, despite these qualities, the precise implementation, effect, and benefit of social media in online weight-management interventions remains unknown.

For these reasons, we conducted a systematic review of the literature to understand whether and how online weight-management interventions have used social media to improve weight-related outcomes, such as weight loss, diet, and physical activity.

## Methods

### Data Sources and Search Terms

We followed the Preferred Reporting Items for Systematic Reviews and Meta-Analyses (PRISMA) recommendations in conducting this systematic review [11]. With the assistance of a research librarian with experience in social media, we searched PubMed, PsycINFO, and EMBASE for articles written in English that reported outcomes associated with the use of social media in online interventions for weight management. Because the Medical Subject Heading (MeSH) term “social media” was not created until 2012, we developed a search strategy that included the following keywords to identify social media: social media, social technology, social network, online community, wiki, YouTube, Facebook, Myspace, Flickr, Twitter, and Delicious. MeSH terms and keywords to represent weight management included obesity, overweight, weight gain, weight loss, body mass index, diet, and physical activity. The full search criteria for PubMed is presented in Multimedia Appendix 1. Additional studies were identified through hand searches of electronically retrieved articles, review articles, and from a cited reference search (Web of Science and Scopus). No limits or filters were placed on search criteria; electronic searches were last updated on March 25, 2013.

### Study Selection and Definitions

Studies were included if they were (1) randomized controlled trials (RCTs); (2) published in peer-reviewed literature; (3), reported weight-related outcomes, such as body mass index (BMI) or weight, dietary intake, or physical activity; and (4) included a social media component. As defined by Kaplan et al [12], we defined social media as Web-based applications that allow individuals to interact in a virtual community by exchanging user-generated information (eg, online discussion board, online bulletin board, chat room, online community). Weight-related outcomes included measures such as BMI, body weight, percent body fat, and waist and hip circumference. We defined devices that measured the intensity of physical activity as locomotion accelerometers, whereas pedometers were defined as devices that specifically measured step count [13].

### Data Extraction

Two authors (TC and SW) independently abstracted variables by using a standardized template. Abstracted data included study variables (recruitment criteria, setting), participant variables (mean age, gender, mean BMI), intervention variables (brief description of weight-management intervention, intervention duration, type of social media used), outcome variables (eg, BMI, waist circumference, physical activity level, dietary intake), and quality variables (eg, data on randomization, control group, isolation of social media component). When encountered, discrepancies were resolved by consensus during a series of face-to-face and email discussions between 2 investigators (TC and SW).

### Risk of Study Bias

The risk for bias in each RCT was assessed using the Jadad scale, which incorporates study domains including randomization, blinding, and description of withdrawals and dropouts [14]. Studies that received 4 or greater out of 5 possible points on the Jadad scale were considered as being at low risk of bias whereas scores of 2 and 3 or 0 and 1 were considered to be at moderate or high-risk of bias, respectively.

In addition, because our main interest was the effect of social media on online weight interventions, study quality was also rated using methodology developed by Norman et al [15]. Based on 9 methodological characteristics, this approach specifically evaluates the impact of technology (eg, social media) on specified outcomes of interest, thus allowing for a more precise approach to measuring these types of interventions. The Norman score also includes assessment of randomization, inclusion of a control group, pre-post test design, retention, baseline group equivalence, missing data, sample size calculations, and the validity of outcome measures. Each study was given 1 point for each criterion present with a maximum score of 9. Studies that scored 7 to 9 were considered high quality, studies that scored 5 to 7 were considered of moderate quality, and scores of <5 were considered poor quality.

### Data Synthesis

Because the included studies varied greatly on study design, participants, measures, outcomes, and social media components, meta-analysis of interventions was not attempted or performed.

## Results

### Overview

**Figure 1:**
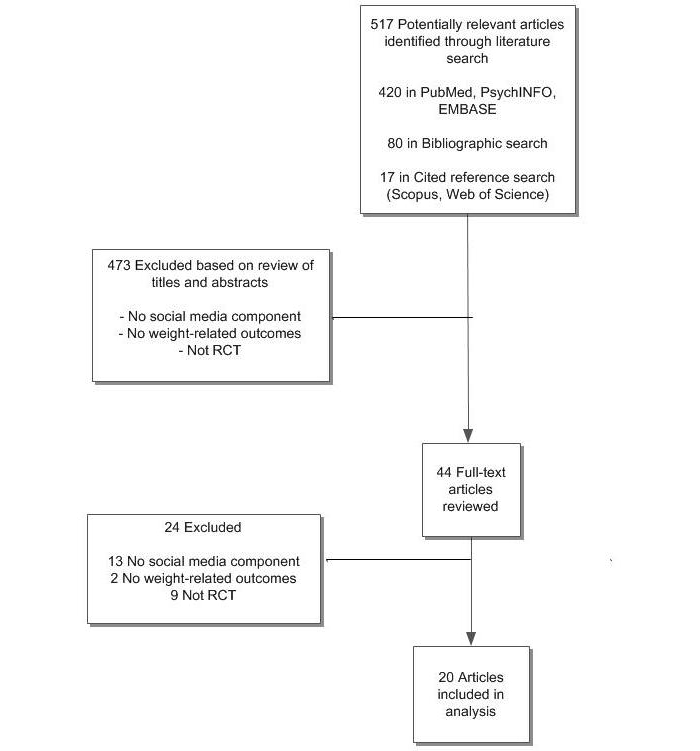
Study flow.

In total, 517 studies were identified by our electronic searches. Following application of eligibility criteria, 20 studies [16-35] met our inclusion criteria for analysis (Figure 1). All 20 included studies involving adult populations and were published between 2001 and 2013. Studies were conducted in various parts of the world, including the United States (n=14), Australia (n=3), Canada (n=2), and the United Kingdom (n=1). Of the included studies, one study focused only on diet [16], 5 studies only on physical activity [17-21], 12 studies on both diet and physical activity [22-33], and 2 studies on weight maintenance after weight loss [34,35] (Table 1). Please see Multimedia Appendix 2 for a table of detailed study characteristics.

**Table 1:** Summary study characteristics.^a^

Source		Population					Type of social media	Primary outcomes	Results	Risk of bias (Jadad scale)	Quality score
		n	RR^b^ (%)	Mean age	% Female	Mean BMI					
**Diet**											
	Verheijden et al (2004) [16]	146	89	63	24.0	29.4	Bulletin board	Social support, BMI, waist-to-hip ratio, blood pressure, and cholesterol levels	No statistically significant differences in outcomes	Low	High
**Physical activity**											
	Hurling et al (2007) [17]	77	100	40.4	66.2	26.3	Chat room–style message board	Change in moderate physical activity	Higher level of moderate physical activity and more percent body fat lost in the test group	Mod	High
	Ferney et al (2009) [18]	106	87.7	52.1	71.7	NR	Bulletin board	Self-reported walking and physical activity	No statistically significant differences in outcomes	Mod	Mod
	Liebreich et al (2009) [19]	49	89.8	54.1	59	33.9	Message board	Self-reported BMI, physical activity, and social cognitive measures	Significant improvement in total vigorous and moderate minutes of physical activity in intervention group	High	Mod
	Richardson et al (2010) [20]	324	76.2	52	65	33.2	Online community with message board	Change in average daily step counts from baseline, valid days of pedometer data, and online community use	No statistically significant differences in outcomes	Mod	High
	Cavallo et al (2012) [21]	134	89.6	NR	100	NR	Facebook	Self-report social support and physical activity	No statistically significant differences in outcomes	High	High
**Diet and physical activity**											
	Tate et al (2001) [25]	91	71.4	40.9	89.0	29.0	Bulletin board	Body weight and waist circumference	Behavior therapy group lost more weight and had greater changes in waist circumference	Mod	Mod
	Tate et al (2003) [26]	92	100	48.5	89.1	33.1	Message boards	Body weight, BMI, and waist circumference	Behavioral e-counseling group had greater reduction in weight, percentage of initial body weight, BMI, and waist circumference	Mod	High
	Womble et al (2004) [32]	47	66.0	43.7	100	33.5	Online meetings, online bulletin board	Weight change	Manual group lost significantly more weight than the eDiets.com intervention	Mod	High
	Tate et al (2006) [27]	192	80.7	49.2	84.4	32.7	Bulletin board	Weight loss, dietary intake, and physical activity	Weight losses were significantly greater in the human email-counseling group than computer-automated feedback or no counseling groups	Mod	High
	Gold et al (2007) [28]	124	71.0	47.7	81.5	32.4	Discussion board, online chats/meetings	Change in body weight	VTrim group lost significantly more weight than the eDiets.com group at 6 months and maintained a greater loss at 12 months	Mod	Mod
	Webber et al (2008) [22]	66	98.5	50	100	31.1	Separate message board for each website group, and online chat	Body weight	Minimal group lost more than the enhanced group	High	High
	Morgan et al (2009) [29]	65	100	35.9	0	30.6	Bulletin board	Weight, waist circumference, BMI	Greater weight loss for the Internet group	Low	High
	Sternfield et al (2009) [30]	787	69.8	40.1	78.1	in categories	Discussion board	Self-reported change in dietary intake and physical activity	Intervention group had increased physical activity, and increased consumption of fruits and vegetables	High	Mod
	Harvey-Berino et al (2010) [31]	481	96.0	46.6	93	35.7	Chat rooms and a bulletin board	Body weight and BMI	Weight loss for InPerson was significantly greater than the Internet and Hybrid conditions	Mod	High
	Turner-McGrievy et al (2011) [23]	96	89.6	42.9	75	32.5	Twitter	Body weight	No statistically significant differences in outcomes	Mod	High
	Brindal et al (2012) [33]	8112	5.2	45.0	83	34.0	Social networking platform: friend networks, blogs, discussion forums, and news feeds	Body weight	No statistically significant differences in outcomes	Low	Mod
	Napolitano et al (2013) [24]	52	96	20.5	86.5	31.4	Facebook	BMI	Facebook Plus group had significantly greater weight loss than Facebook and waiting list	High	High
**Weight maintainence**											
	Harvey-Berino et al (2004) [34]	255	69	45.8	82	31.8	Chat room and bulletin board	Body weight, height, energy intake, and energy expended	No statistically significant differences in outcomes	Mod	High
	Cussler et al (2008) [35]	161	69	48	100	31.1	Bulletin board and chat rooms	BMI, body fat percentage, and total body fat mass	No statistically significant differences in outcomes	High	Mod

^a^For detailed study characteristics, risk of bias, and quality scores, please see Multimedia Appendix 2.

^b^RR: response rate.

### Diet Interventions

Only one study (n=146) [16] focused solely on a dietary intervention for weight management. This study tested whether Web-based nutrition counseling and a social support tool that included a bulletin board could improve weight outcomes. Low uptake of the Web-based intervention (24 of 73 participants) with limited posting on the bulletin board was reported. Messages on the bulletin board mostly contained requests for factual information directed to the research team with minimal participant interaction. The study found no significant differences between the intervention group and the usual care arm for any outcome [16].

### Physical Activity Interventions

Five studies featured interventions targeting physical activity (n=690) [17-21]. These studies tested websites with a variety of other components. One study used accelerometers plus a website, one study used a pedometer plus a website, 2 interventions included only a website, and 1 used a website plus Facebook. Excluding the study that used Facebook, the social media component for all other studies in this category were message boards within the intervention website.

Only one study specifically isolated and measured the effect of the social media component, by including it in only 1 arm (online community) of the study [20]. Although this study found no difference in physical activity among the groups, the percentage of participants that completed the study and length of engagement was greater for those randomized to the social media component (ie, the online community).

Among the remaining 4 studies, 2 reported the usage of the social media component [17,18]. Within the 2 studies that reported social media use, Hurling et al [17] found that the chat room-style message board was the most frequently used component. In contrast, Ferney et al [18] reported that only 1 message was posted on their bulletin board and hypothesized that this was because of the small number of participants enrolled in the study (n=52). Although the study by Liebreich et al [19] did not report data on message board use, the authors theorized that the message board encouraged interactivity and, thus, adherence. With respect to weight outcomes, Cavallo et al [21] used Facebook as an adjunct for social support between participants and found no increased self-reported social support or physical activity. However, the remaining 3 studies showed higher levels of physical activity or greater maintenance of physical activity in participants in the intervention arms [17-19].

### Diet and Physical Activity Interventions

Twelve studies featured interventions that included both diet and physical activity components (n=10,205) [22-33]. In addition to the typical online intervention and counseling, Tate et al [27] also included structured meals and meal replacements.

Most interventions in this category featured bulletin/message boards, chat rooms, or both as their social media component. Tate et al [27] created an online “ebuddy” tool that matched participants with others with similar characteristics across the country to gain support. In contrast, Turner-McGrievy et al [23] and Napolitano et al [24] used available mainstream social media, such as Twitter and Facebook, for education and to provide support to participants. Despite the use of social media in these 12 studies, no study uniquely isolated the effect of these platforms on participants; rather, the featured bulletin boards and chat rooms were embedded within a larger intervention.

Data regarding the frequency of use of the social media component were rarely reported, although when it was, use was low. For example, Tate et al [25] found that only 28% of participants ever posted a note to a bulletin board (range 1-7 postings per person) over 6 months. Examining the popularity of postings, Napolitano et al [24] found that less than one-quarter of the participants “liked” the study-related posts on Facebook.

Although the correlation between social media use and weight loss was generally positive, it was only reported in a few studies and could be because of greater adherence to the interventions overall. In the studies by Gold et al [28] and Webber et al [22], weight loss was correlated with bulletin board use in both arms. Likewise, Turner-McGrievy et al [23] found that the number of weight loss podcasts downloaded over 6 months was significantly correlated with weight loss in both arms of the study.

Although the influence of social media on weight-related measures was not specifically tested in any of these studies, findings were heterogeneous. For instance, 2 studies reported positive outcomes (greater weight loss, increased physical activity, increased consumption of fruits and vegetables, and marginally decreased sugar intake) in those randomized to interventions containing social media [29,30]. Conversely, 2 studies reported less weight loss in the study arm that included the social media component [31,32]. Other studies either had social media components in multiple arms of the study (n=7) [22-28] or showed no difference in weight outcomes (n=1) [33].

### Weight Maintenance Interventions

Two studies (n=416) featured interventions focused on weight maintenance after weight loss. The social media components of the online weight maintenance interventions included both online bulletin boards and chat rooms. Overall, inclusion of social media did not result in differences in weight outcomes. In the study by Harvey-Berino et al [34], the arm with social media demonstrated no difference in perceived support compared to in-person therapy and it also had the highest rates of attrition. Interestingly, 100% of the participants within the social media arm in one study contributed to the bulletin board of the website, demonstrating high engagement with the social media component [35].

### Risk of Study Bias

The median Jadad score overall was 2 out of 5 points (median 2, range 1-5) representing moderate risk for bias in the included studies (Table 1). Because many studies were unable to blind participants’ and/or study coordinators’ participation in social media, all but 2 studies had 2 of 5 points deducted for not describing a double-blinding process.

Using the scale developed by Norman et al [15], the median study score was 7 out of 9 (range 6-8, median 7) representing overall high study quality (Table 1). Only one study isolated social media in the design of their intervention, and 9 studies (45%) did not report a rationale for sample size. The median retention rate was 88.4% (range 5%-100%). Please see Multimedia Appendices 3 and 4 for detailed risk of bias and quality scoring data for each study.

## Discussion

### Principal Findings

In our systematic review of RCTs evaluating online weight-management interventions, we found that few studies implemented social media in a manner in which its impact could be measured and assessed. Therefore, the effect of social media is difficult to ascertain in the available literature. Our findings are consistent with previous systematic reviews on Internet-based behavioral interventions and electronic peer-to-peer support group interventions, which have found that the effect of the technology being studied was not isolated; thus, their effectiveness is not known [36-40]. Nevertheless, we found that contemporary studies continue to include online support-based behavioral interventions for weight management despite little evidence of their effectiveness.

However, some salient points emerged from the only study in our review that isolated its social media component from a broader intervention [20]. This study found no differences in physical activity outcomes between participants who had access to social media versus those who did not. Among those in the social media arms, greater use of the social media component was associated with improved weight-related outcomes. Therefore, for some people, social media components may be effective in promoting behavior change. Whether it would be effective just for those who are inclined to use it, or whether it would work broadly if one could encourage a wider group of participants to use it, is unknown. However, it appears that social media may fill a gap for some participants. Specifically, this study found that those with less baseline social supports (ie, family, friends) were more likely to use the social media component and that greater use of the social media component among this group was associated with lower dropout rates. This finding is consistent with other studies that suggested that use of social networking sites helped to satisfy the need for social support and connectedness [9,10].

We also observed that social media was incorporated into online interventions largely through the use of discussion boards and chat rooms. Mainstream social media platforms (eg, Facebook, Twitter) were used in only 15% of studies and mainly in more recent publications (2011-2013). This may indicate a move from program-specific, investigator-developed interventions to those that capitalize on media that participants already frequent. Furthermore, the extent of actual social media use in these studies was inconsistently reported and when reported, use was mostly low.

Why has social media not had as much uptake in weight-based interventions compared to other areas of life? One reason for this disparity may be the artificial nature of the types of social media (discussion boards and chat rooms) used on websites developed for weight management. The majority of current mainstream social media use relies on sophisticated, user-friendly, vibrant platforms that incorporate a rich, pleasing, graphical environment allowing for instantaneous transfer of information to a large community of users. Conversely, the components designed for weight-management studies may not have the same usability, access, or appeal. Furthermore, although the majority of Americans associate social media with positive terms such as good, great, fun, interesting, and convenient, the use of social media for weight management may diminish these positive feelings by associating its use with a health-specific and sensitive condition: weight management [41].

Studies often reported that social media components were included to encourage support from other participants and to build community, although no study reported increased levels of social support after use of the social media components. A possible explanation relates to how social media has evolved over the years. Social media began as virtual communities and computer-mediated communication, which was based on the assumption that people participating would be using these platforms to connect with new people who shared similar interests or life experiences [42]. Current social networking sites can be distinguished from these early virtual communities by the fact that they are primary used for the conversion and maintenance of existing relationships into online ones [41,43]. Therefore, social support through social media platforms currently being employed by online interventions may simply be hampered as a result of this stranger phenomenon, a hypothesis supported by the fact that 57% of Americans explicitly report that they do not use social media to make new acquaintances [41]. One plausible strategy to overcome this weakness may be to supplement online interventions with face-to-face interventions. Incorporating this traditional way to cultivate relationships with the use of online social media is more in-line with how social media is used today.

### Limitations

Our systematic review has some limitations. First, outcomes varied within the included studies so that studies could not be analyzed together or compared with one another. Second, most studies did not isolate the unique impact of social media on weight outcomes; thus, the role of social media in these interventions remains unknown. Third, risk of bias and study quality varied considerably within the included studies. Fourth, social media applications and platforms are evolving rapidly and it is possible, despite a rigorous search strategy, that studies of certain mobile devices with social media capabilities will be missed by our review. Finally, we limited our inclusion to RCTs only; other study designs may have been used to examine the use of this relatively novel technology in weight management.

### Conclusions

Despite these limitations, our systematic review provides a comprehensive review of how social media is being used in online weight-management interventions to date. We found that social media is being incorporated in online weight-management interventions largely through message boards and chat rooms with unclear benefits. Although social media may play a role in retaining and engaging participants in online weight loss interventions, studies that are designed to measure the effect of social media are needed to understand whether and how social media may meaningfully improve weight management.

## Multimedia Appendix 1

Full PubMed search strategy.

## Multimedia Appendix 2

Detailed study characteristics.

## Multimedia Appendix 3

Jadad scale.

## Multimedia Appendix 4

Quality scores.

## Abbreviations

BMI: body mass index
MeSH: Medical Subject Heading
PRISMA: Preferred Reporting Items for Systematic Reviews and Meta-Analyses
RCT: randomized controlled trial

## References

[1] Finkelstein EA, Trogdon JG, Cohen JW, Dietz W (2009). Annual medical spending attributable to obesity: payer- and service-specific estimates. Health Aff (Millwood).

[2] Woolford SJ, Gebremariam A, Clark SJ, Davis MM (2009). Persistent gap of incremental charges for obesity as a secondary diagnosis in common pediatric hospitalizations. J Hosp Med.

[3] Woolford SJ, Gebremariam A, Clark SJ, Davis MM (2007). Incremental hospital charges associated with obesity as a secondary diagnosis in children. Obesity (Silver Spring).

[4] Freedman DS, Dietz WH, Srinivasan SR, Berenson GS (1999). The relation of overweight to cardiovascular risk factors among children and adolescents: the Bogalusa Heart Study. Pediatrics.

[5] Tsiros MD, Olds T, Buckley JD, Grimshaw P, Brennan L, Walkley J, Hills AP, Howe PR, Coates AM (2009). Health-related quality of life in obese children and adolescents. Int J Obes (Lond).

[6] Hill AJ, Silver EK (1995). Fat, friendless and unhealthy: 9-year old children's perception of body shape stereotypes. Int J Obes Relat Metab Disord.

[7] Fakhouri TH, Ogden CL, Carroll MD, Kit BK, Flegal KM (2012). Prevalence of obesity among older adults in the United States, 2007-2010. NCHS Data Brief.

[8] Madden M, Zickuhr K (2011). 65% of online adults use social networking sites.

[9] Manago AM, Taylor T, Greenfield PM (2012). Me and my 400 friends: the anatomy of college students' Facebook networks, their communication patterns, and well-being. Dev Psychol.

[10] Köbler F, Riedl C, Vetter C, Leimeister JM, Krcmar H (2010). Social connectedness on Facebook- An explorative study on status message usage. Proceedings of the Sixteenth Americas Conference on Information Systems, Lima, Peru, August 12-15.

[11] Moher D, Liberati A, Tetzlaff J, Altman DG, PRISMA Group (2009). Preferred reporting items for systematic reviews and meta-analyses: the PRISMA statement. J Clin Epidemiol.

[12] Kaplan AM, Haenlein M (2010). Users of the world, unite! The challenges and opportunities of Social Media. Business Horizons.

[13] Welk GJ, McClain J, Ainsworth BE (2012). Protocols for evaluating equivalency of accelerometry-based activity monitors. Med Sci Sports Exerc.

[14] Jadad AR, Moore RA, Carroll D, Jenkinson C, Reynolds DJ, Gavaghan DJ, McQuay HJ (1996). Assessing the quality of reports of randomized clinical trials: is blinding necessary?. Control Clin Trials.

[15] Norman GJ, Zabinski MF, Adams MA, Rosenberg DE, Yaroch AL, Atienza AA (2007). A review of eHealth interventions for physical activity and dietary behavior change. Am J Prev Med.

[16] Verheijden M, Bakx JC, Akkermans R, van den Hoogen H, Godwin NM, Rosser W, van Staveren W, van Weel C (2004). Web-based targeted nutrition counselling and social support for patients at increased cardiovascular risk in general practice: randomized controlled trial. J Med Internet Res.

[17] Hurling R, Catt M, Boni MD, Fairley BW, Hurst T, Murray P, Richardson A, Sodhi JS (2007). Using internet and mobile phone technology to deliver an automated physical activity program: randomized controlled trial. J Med Internet Res.

[18] Ferney SL, Marshall AL, Eakin EG, Owen N (2009). Randomized trial of a neighborhood environment-focused physical activity website intervention. Prev Med.

[19] Liebreich T, Plotnikoff RC, Courneya KS, Boulé N (2009). Diabetes NetPLAY: A physical activity website and linked email counselling randomized intervention for individuals with type 2 diabetes. Int J Behav Nutr Phys Act.

[20] Richardson CR, Buis LR, Janney AW, Goodrich DE, Sen A, Hess ML, Mehari KS, Fortlage LA, Resnick PJ, Zikmund-Fisher BJ, Strecher VJ, Piette JD (2010). An online community improves adherence in an internet-mediated walking program. Part 1: results of a randomized controlled trial. J Med Internet Res.

[21] Cavallo DN, Tate DF, Ries AV, Brown JD, DeVellis RF, Ammerman AS (2012). A social media-based physical activity intervention: a randomized controlled trial. Am J Prev Med.

[22] Webber KH, Tate DF, Michael Bowling J (2008). A randomized comparison of two motivationally enhanced Internet behavioral weight loss programs. Behav Res Ther.

[23] Turner-McGrievy G, Tate D (2011). Tweets, Apps, and Pods: Results of the 6-month Mobile Pounds Off Digitally (Mobile POD) randomized weight-loss intervention among adults. J Med Internet Res.

[24] Napolitano MA, Hayes S, Bennett GG, Ives AK, Foster GD (2013). Using Facebook and text messaging to deliver a weight loss program to college students. Obesity (Silver Spring).

[25] Tate DF, Wing RR, Winett RA (2001). Using Internet technology to deliver a behavioral weight loss program. JAMA.

[26] Tate DF, Jackvony EH, Wing RR (2003). Effects of Internet behavioral counseling on weight loss in adults at risk for type 2 diabetes: a randomized trial. JAMA.

[27] Tate DF, Jackvony EH, Wing RR (2006). A randomized trial comparing human e-mail counseling, computer-automated tailored counseling, and no counseling in an Internet weight loss program. Arch Intern Med.

[28] Gold BC, Burke S, Pintauro S, Buzzell P, Harvey-Berino J (2007). Weight loss on the web: A pilot study comparing a structured behavioral intervention to a commercial program. Obesity (Silver Spring).

[29] Morgan PJ, Lubans DR, Collins CE, Warren JM, Callister R (2009). The SHED-IT randomized controlled trial: evaluation of an Internet-based weight-loss program for men. Obesity (Silver Spring).

[30] Sternfeld B, Block C, Quesenberry CP, Block TJ, Husson G, Norris JC, Nelson M, Block G (2009). Improving diet and physical activity with ALIVE: a worksite randomized trial. Am J Prev Med.

[31] Harvey-Berino J, West D, Krukowski R, Prewitt E, VanBiervliet A, Ashikaga T, Skelly J (2010). Internet delivered behavioral obesity treatment. Prev Med.

[32] Womble LG, Wadden TA, McGuckin BG, Sargent SL, Rothman RA, Krauthamer-Ewing ES (2004). A randomized controlled trial of a commercial internet weight loss program. Obes Res.

[33] Brindal E, Freyne J, Saunders I, Berkovsky S, Smith G, Noakes M (2012). Features predicting weight loss in overweight or obese participants in a web-based intervention: randomized trial. J Med Internet Res.

[34] Harvey-Berino J, Pintauro S, Buzzell P, Gold EC (2004). Effect of internet support on the long-term maintenance of weight loss. Obes Res.

[35] Cussler EC, Teixeira PJ, Going SB, Houtkooper LB, Metcalfe LL, Blew RM, Ricketts JR, Lohman J, Stanford VA, Lohman TG (2008). Maintenance of weight loss in overweight middle-aged women through the Internet. Obesity (Silver Spring).

[36] Manzoni GM, Pagnini F, Corti S, Molinari E, Castelnuovo G (2011). Internet-based behavioral interventions for obesity: an updated systematic review. Clin Pract Epidemiol Ment Health.

[37] Eysenbach G, Powell J, Englesakis M, Rizo C, Stern A (2004). Health related virtual communities and electronic support groups: systematic review of the effects of online peer to peer interactions. BMJ.

[38] Neve M, Morgan PJ, Jones PR, Collins CE (2010). Effectiveness of web-based interventions in achieving weight loss and weight loss maintenance in overweight and obese adults: a systematic review with meta-analysis. Obes Rev.

[39] Lau PW, Lau EY, Wong del P, Ransdell L (2011). A systematic review of information and communication technology-based interventions for promoting physical activity behavior change in children and adolescents. J Med Internet Res.

[40] van den Berg MH, Schoones JW, Vliet Vlieland TP (2007). Internet-based physical activity interventions: a systematic review of the literature. J Med Internet Res.

[41] Smith A (2011). Why Americans use social media.

[42] Wellman B, Salaff J, Dimitrova D, Garton L, Gulia M, Haythornthwaite C (1996). Computer networks as social networks: Collaborative work, telework, and virtual community. Annu Rev Sociol.

[43] Ellison NB, Steinfield C, Lampe C (2007). The benefits of Facebook "friends": Social capital and college students' use of online social network sites. J Comput-Mediat Comm.

