# Supplementary material for: The Role of Social Media in Online Weight Management: Systematic Review
Source: J Med Internet Res. 2013 Nov 28;15(11):e262. doi: 10.2196/jmir.2852 (PMC3868982; doi:10.2196/jmir.2852)
Supplement: Supplementary file 1 [file jmir_v15i11e262_app1.pdf]

Appendix A. Full PubMed search criteria

("social media"[TIAB] OR "social bookmarking"[TIAB] OR "social technology"[TIAB] OR "social technologies"[TIAB] OR "folksonomy"[TIAB] OR "folksonomies"[TIAB]) OR ("mashup"[TIAB] OR "mashups"[TIAB]) OR (wikis[TIAB] OR wiki[TIAB] OR wikipedia[TIAB]) OR blog*[TIAB] OR ("social networking"[TIAB] OR "social networks"[TIAB] OR "social network"[TIAB] OR "online community"[TIAB] OR "online communities"[TIAB] OR facebook[TIAB] OR myspace[TIAB] OR ning[TIAB]) OR ((youtube[TIAB]) OR "flickr"[TIAB] OR (twitter[TIAB] OR tweet[TIAB]) OR (delicious[TIAB])))

AND

((Obesity[MeSH]) OR (Weight gain[MeSH]) OR (Weight loss[MeSH]) OR (Body mass index[MeSH]) OR (skinfold thickness[MeSH]) OR (waist-hip ratio[MeSH]) OR (abdominal fat[MeSH]) OR (overweight[MeSH]) OR (overweight*[TIAB] OR overweight[TIAB] OR over-weight[TIAB]) OR ("fat overload"[TIAB] AND syndrom*[TIAB]) OR (overeat*[TIAB] OR over-eat*[TIAB]) OR (overfeed*[TIAB] OR over-feed*[TIAB]) OR (adipos*[TIAB]) OR (obes*[TIAB]) OR ("body mass index"[TIAB] OR "body mass indices"[TIAB] OR "body mass indexes"[TIAB] OR BMI[TIAB]) OR (waist-hip[TIAB] AND ratio*[TIAB]) OR (skinfold[TIAB] AND thickness*[TIAB]) OR (abdominal[TIAB] AND fat*[TIAB]) OR weight[TIAB] OR (Metabolic Syndrome X[MeSH] OR "metabolic syndrome"[TIAB]) OR ("bariatric surgery"[MeSH Terms] OR "bariatric surgery"[TIAB])) OR "Motor Activity" [Mesh] OR "sports" [Mesh] OR "physical fitness" [Mesh] OR "physical exertion" [Mesh] OR "physical activity" [All Fields] OR "exercise" [All Fields] OR "walking" [Mesh]) OR “diet” [All Fields] OR “food” [MeSH] OR “hunger” [MeSH]
