# Supplementary material for: The Role of Social Media in Online Weight Management: Systematic Review
Source: J Med Internet Res. 2013 Nov 28;15(11):e262. doi: 10.2196/jmir.2852 (PMC3868982; doi:10.2196/jmir.2852)
Supplement: Supplementary file 2 [file jmir_v15i11e262_app2.pdf]

Appendix B. Study Characteristics							
**Source**	**Population**	**Setting**	**Intervention**	**Control condition**	**Type of Social Media**	**Outcomes and Measures**	**Results**
**DIET**							
Verheijden et al. (2004)	n= 146<br>Retention Rate=89%<br>Mean Age= 63 (10.5)<br>24.0% female<br>Mean BMI= 29.4 (4.9)<br>Patients 40 years and older who appeared in the computerized billing system in the year before recruitment for at least 1 of the following: hypertension, type 2 diabetes mellitus, and dyslipidemia	Canada	Usual healthcare plus access to a Web-based nutrition counselling and social support tool (Heartweb), which targets counselling messages to patient's readiness to decrease fat consumption and includes a bulletin board for patients to post messages for social support.	Usual healthcare only.	Bulletin Board	Social support, BMI, waist-to-hip ratio, blood pressure, and cholesterol levels measured at 0, 4, and 8 months.	Intention-to-treat found no statistically significant differences between groups with respect to change in any outcome measurement from baseline to 4 and 8 months. Patient-patient interaction via the bulletin board was limited.
**PHYSICAL ACTIVITY**							
Hurling et al. (2007)	n= 77<br>Retention Rate=100%<br>Mean Age= 40.4 (7.6)<br>66.2% female<br>Mean BMI=26.3 (3.4)<br>Age 30 to 55 years; BMI 19 to 30; not vigorously active; not taking regular prescription medication	United Kingdom	Bluetooth Actiwatch Accelerometers and access to an internet-based behavioral change system for 9 weeks. Participants could use the system to determine their perceived barriers, report weekly physical activity, receive constructive feedback, have access to weekly schedules for planning physical activity, and could choose to receive email or text reminders.	Bluetooth Actiwatch Accelerometers and verbal advice about recommended physical activity levels, but no feedback or website access.	Chatroom style message board in interventio n group	Primary: Change in moderate physical activity before and after 9 week intervention.<br>Secondary: Changes in weight, percent body fat, height, and resting blood pressure.	Intention-to-treat analyses found a higher level of moderate physical activity and more percent body fat lost in the test group compared to control group.
Ferney et al. (2009)	n= 106<br>Retention Rate=87.7%<br>Mean Age= 52.1 (4.6)<br>71.7% female<br>Mean BMI=not reported<br>Age 45- 60 years; inactive	Australia	Get Up And Go: a local neighborhood environment-focused website intervention that includes sections for cognitive and behavioral strategies, examples of activities, links to relevant websites, a tool for self-monitoring and goal-setting, a searchable database for local opportunities for physical activity, a calendar of events, a map of walking trails, physical activity profiles for each suburb, news items, and a bulletin board. Intervention participants received tailored emails.	Active Living Online: motivational-information website intervention with sections corresponding to the motivational stages of change, and sequential movement through each section. Control participants received the same number of emails as the intervention group, though messages were non-tailored.	Bulletin Board in interventio n group	Primary: Self reported-walking and physical activity was determined at weeks 0, 12, and 26.<br>Secondary: Program use through background programming and self-reported use of website programs was determined at week 12.	Intention-to-treat analyses showed that both groups showed increase in physical activity and walking. Website use was significantly greater among intervention participants. Intervention group participants maintained more of their initial increase in physical activity at week-26. Those in the intervention group who used the website more reported significantly more walking along the community trail at week-26.
Liebreich et al. (2009)	n= 49<br>Retention Rate=89.8%<br>Mean Age= 54.1 (10.3)<br>59% female<br>Mean BMI= 33.9 (7.4)<br>Age 18 and older; has type 2 diabetes	Canada	Diabetes NETPLAY program: a website comprised of sections with a weekly topic, education, research, fitness tips, physical activity myths, a physical activity logbook, message board and weekly email counselling from the study coordinator.	Control group received static links to the Canadian Diabetes Association's Clinical Practice Guidelines for physical activity and Health Canada's Physical Activity Guide.	Message board in interventio n group	Self-reported BMI, physical activity and social cognitive measures were measured at 0 and 12 weeks.	Intention-to-treat analysis showed that the intervention group demonstrated a significant improvement in total vigorous and moderate minutes of physical activity compared to the control group.
Richardson et al. (2010)	n= 324<br>Retention Rate=76.2%<br>Mean Age= 52 (11.4)<br>65% female<br>Mean BMI= 33.2 (6.2)<br>Age 18 and older; sedentary; has either BMI ≥ 25, type 2 diabetes, or coronary artery disease	US	Stepping up to Health (SUH) internet walking program: includes pedometers, step-count feedback, individually assigned and gradually incrementing step-count goals, and individually tailored motivational messages. Also had access to an online community and were encouraged to post self-introductions and messages modeling self-regulation strategies such as overcoming barriers and describing successes.	Stepping up to Health (SUH) internet walking program only. No access to online community features.	Online community with message board in interventio n group only	Change in average daily step counts from baseline, valid days of pedometer data, and online community use.	Both groups showed increases in average daily step counts at the end of the intervention period, but there were no significant differences in increase step counts between arms using either intention-to-treat or completers analysis. The intervention group showed a higher percentage of completers and remained engaged longer than the control group.
Cavallo et al. (2012)	n=134<br>Retention Rate= 89.6%<br>Mean Age= not reported<br>100% female<br>Mean BMI= not reported<br>Undergraduate students age <25; reported <30 min of daily physical activity; reported >30 min of daily use of Facebook	US	Internet Support for Healthy Associations Promoting Exercise (INSHAPE) website: includes educational information and self-monitoring tools. Intervention group also invited to private Facebook group to exchange social support.	Limited version of INSHAPE website: excluded self-monitoring, and received emails with same news stories provided to Facebook group.	Facebook	Primary: Self-report social support at baseline and 12 weeks.<br>Secondary: Self-report physical activity at baseline and 12 weeks.	Intention-to-treat analysis found no differences in perceived social support or physical activity between groups over time.
**DIET AND PHYSICAL ACTIVITY**							

Tate et al. (2001)	n= 91<br>Retention Rate=71.4%<br>Mean Age= 40.9 (10.6)<br>89.0% female<br>Mean BMI= 29.0 (3.0)<br>Healthy, overweight adult hospital employees age 18 to 60 years with BMI 25-36	US	All participants received a one hour lesson on behavioral weight control. Internet behavioral therapy group had access to website, instruction to report weight, calories, fat grams, and exercise energy expenditure each week via electonic diary accessible on study website, 24 weekly email messages including structural guidance on nutrition, exercise, and behavioral self-regulatory strategies, and individualized feedback with recommendations and reinforcement, as well as access to electronic bulletin board.	Access to study website only. No email messages.	Bulletin board	Body weight and waist circumference measured at 0, 3, and 6 months.	Repeated measures analysis showed behavior therapy group lost more weight and had greater changes in waist circumference than control group.
Tate et al. (2003)	n=92<br>Retention Rate=100%<br>Mean Age=48.5 (9.4)<br>89.1% female<br>Mean BMI= 33.1 (3.8)<br>Overweight/obese adults; has BMI 27-40; has 1 or more risk factor for type 2 diabetes	US	All participants attended a 1 hr standard weight control instruction on diet, exercise, and behavior change. Intervention participants were enrolled in an internet weight loss program and received behavioral counseling via email 5x/week for one month, then weekly for the remaining 11 months.	Basic internet weight loss program with separate message board, and weekly email reminders to submit weight information. No behavioral counseling.	Separate message boards	Body weight, waist circumference, and fasting blood glucose 0, 3, 6, and 12 months.	Intention-to-treat analysis at 12 months showed behavioral e-counseling group had greater reduction in weight (-4.4 kg), percentage of initial body weight, BMI, and waist circumference.
Womble et al. (2004)	n=47<br>Retention Rate=66.0%<br>Mean Age= 43.7 (10.2)<br>100% female<br>Mean BMI= 33.5 (3.1)<br>Healthy women age 18-65 with BMI 27-40	US	eDiets.com: website that provided a diet to fit their lifestyle, a vitual visit with a dietitian, social support with on-line meetings moderated by a professional, on-line bulletin board support groups, animated fitness instructor, email reminders about program and their goals, biweekly diet and fitness email newsletters, and a "find a buddy" system. Both groups met with psychologist at 0, 8, 16, 26, 52 weeks to review goals and methods of treatment, address progress and satisfaction.	Weight loss manual (LEARN Program for Weight Management 2000), a 243 page book that provided 16 step-by-step lessons for modifying eating, activity, and thinking habits. After 16 weeks, participants were given the Weight Maintenance Survival Guide.	Online meetings, online bulletin board support groups	Primary: Weight change at weeks 0, 2, 4, 8, 12, 16, 20, 26, 34, 42, and 52. Secondary: Blood pressure, triglycerides, glucose, total cholesterol, HDL, LDL obtained at weeks 0, 16, 52. Mood assessed by Beck Depression Inventory-II, quality of life evaluated by Medical Outcomes Study, Short Form-36 Health Survey, eating behavior assessed by Eating Inventory at weeks 0, 16, 52, and Behavioral Adherence in first 16 weeks assessed by completion of food diaries weekly.	LOCF analysis found that women in the manual group lost significantly more weight than the eDiets.com intervention, while there were no significant differences between groups in changes in cardiovascular risk factors, quality of life, or attrition (both 34%).
Tate et al. (2006)	n=192Retention Rate= 80.7%Mean Age= 49.2 (9.8)84.4% femaleMean BMI= 32.7 (3.5)Healthy adults age 20-65 years with BMI 27-40	US	All participants attended 1 group face-to-face session introducing behavioral weight loss recommendations for diet, exercise, and behavioral change. All participants were instructed about the use of structured meals and meal replacements (Slim-Fast). All participants were instructed on how to use the Slim-Fast Web site that includes weekly graphs of weight, prompts to report weight, weight loss tips via email, recipes, and weight loss e-buddy network system. Both e-counseling groups had access to a separate website that offered an electronic diary for weight, caloric intake, use of meal replacements, and exercise, and a message board to post messages to participants in their arm of study. The difference between the two e-counseling groups is that the automated group received automated, but tailored responses based on Cognitive Behavioral Theory, while the other group received email from human weight loss counselor.	Slim-Fast meal replacements and website. No counseling.	Bulletin board	Primary: Weight loss, dietary intake, and physical activity at 0, 3 and 6 months. Secondary: Log-in frequency and website use.	Intention-to-treat analyses showed computer-automated feedback and human email counseling groups had significantly greater weight losses compared with the no counseling group, and these groups did not differ from each other at 3 months. At 6 months, weight losses were significantly greater in the human email counseling group than computer-automated feedback or no counseling groups.
Gold et al. (2007)	n= 124<br>Retention Rate= 71.0%<br>Mean Age= 47.7 (10.4)<br>81.5% female<br>Mean BMI= 32.4 (4.1)<br>Healthy adults over 18 years old with BMI 25-40	US	Vtrim: a 6 month online therapist-led weight loss program that included behavioral strategies and self-management skills on diet and exercise including a one-hour weekly chat and a peer-to-peer discussion board to encourage interaction and group support. Then they participated in a subsequent 6 month online weight maintenance program that included the same features online but less frequently (bi-weekly vs weekly chats).	eDiets: 12 months of self-guided diet and exercise program with automated feedback, professional-facilitated online meetings, chatrooms, discussion boards with hundreds of topics, an archive of frequently asked questions (with expert responses), and a mentor section where new members could team up with a more experienced member.	Discussion board on both websites, online chats/meetings	Primary: Change in body weight at 0, 6, 12 months. Secondary: Dietary intake, physical activity, perceived social support, and website use.	Repeated-measures analyses showed that the VTrim group lost significantly more weight than the eDiets.com group at 6 months and maintained a greater loss at 12 months. More participants in the VTrim group maintained a 5% weight loss goal at 12 months.
Webber et al. (2007)	n=66<br>Retention Rate= 98.5%<br>Mean Age =50 (9.9)<br>100% female<br>Mean BMI= 31.1 (3.7)<br>Healthy adult women age 22-65 with BMI 25-40	US	All participants were provided with a calorie book and self-monitoring diaries, and one separate, but equivalent, intial face-to-face weight loss session including information on exercise and dietary goals performed with motivational interviewing style.<br>Enhanced: Website with weekly lesson plan, message board, links to self-help diet, exercise, and	Minimal: Same website as Enhanced EXCEPT no weekly online chat.	Separate message board for each website group, and online chat for Enhanced website	Primary: Body weight at baseline and 4 months. Secondary: Dietary intake, physical activity, psychosocial variables, and self-monitoring.	Both groups lost weight over time, though group by time interaction was non-signficant. Minimal group lost more than the Enhanced group. Greater program utilization was associated with greater weight loss in both

			behavioral modification resources online PLUS a moderated one-hour weekly online chat.				groups.
Morgan et al. (2009)	n=65<br>Retention Rate= 100%<br>Mean Age= 35.9 (11.1)<br>0% female<br>Mean BMI= 30.6 (2.8)<br>Healthy male staff and students age 18-60 with BMI 25-37	Australia	SHED-IT Internet group: One face-to-face information session (75min) led by researcher plus 3 months of online support on website, Calorie King, which includes an online bulletin board. Participants also received personalized feedback from researchers by email.	Control group attended one information session and received informational booklet identical to Internet group.	Bulletin board on Calorie King	Weight, waist circumference, BMI, blood pressure, resting heart rate, objectively measured physical activity, and self-reported total daily kilojoules assessed at baseline, 3 and 6 months.	Intention to treat analysis revealed greater weight loss for the internet group then for the control group. A significant time effect was found for all outcomes but no between-group differences. Per-protocol analysis revealed significant group-by-time interaction with compliers losing more weight at 6 months than noncompliers and the control group.
Sternfeld et al. (2009)	n= 787<br>Retention Rate= 69.8%<br>Mean Age= 40.1 (10.6)<br>78.1% female<br>Mean BMI =(in categories)<br>Regional employees of Northern California Kaiser Permanente (staff)- no exclusion criteria	US	ALIVE: 16 week tailored email program with a website designed to increase consumption of fruits and vegetables and physical activity and to decrease the consumption of saturated fats, trans fats, and added sugars.	No details reported on control group.	Discussion board on ALIVE website	Self-reported change in dietary intake and physical activity recorded pre-intervention, immediate post-intervention, and 4-month post-intervention.	Intention-to-treat analyses showed the intervention group had increased physical activity, increased consumption of fruits and vegetables, and marginally decreased consumption of sugar. Differences between intervention and control groups were still observed 4 months after the intervention.
Harvey-Berino et al. (2010)	n= 481<br>Retention Rate= 96.0%<br>Mean Age= 46.6 (9.9)<br>93% female<br>Mean BMI= 35.7 (5.6)<br>Healthy adults with BMI 25-50	US	Internet condition: 6 months of weight loss treatment program with weekly group online chat room sessions with homework and online journaling, and access to an online database to help monitor calorie intake (Calorie King).<br>Hybrid condition: Same as Internet condition, though one weekly meeting a month was in-person.	In-person: 6 months of weight loss treatment program focused on the modification of eating and exercise habits with homework assignments, paper journaling, and weekly in-person group session.	Chatrooms and a bulletin board on Calorie King	Primary: Body weight (kg) and BMI at baseline and 6 months.<br>Secondary: Behavior (dietary intake and physical activity), process data (session attendance and journal submissions), and social support (perceived social support scale) at baseline and 6 months.	Intention-to-treat analyses showed weight loss for InPerson was significantly greater than the Internet and Hybrid conditions.
Turner McGrievy et al. (2011)	n= 96<br>Retention Rate= 89.6%<br>Mean Age= 42.9 (11.2)<br>75% female<br>Mean BMI= 32.5 (4.7)<br>Healthy adults age 18-60 with BMI 25-45	US	Both groups received 2 podcasts per week for 3 months and 2 minipodcasts per week for the subsequent 3 months.<br>Podcast group: Received a book with information to monitor their dietary intake.<br>Podcast + Mobile group: Instructed to download a diet and physical activity monitoring app (FatSecret's Calorie counter app, FatSecret.com) AND Twitter to read messages from study coordinator and post at least daily to Twitter.	Please see interventions. No other control group.	Twitter was used by Podcast+ Mobile group	Primary: Body weight at baseline, 3 and 6 months. Secondary: Diet, physical activity, psychosocial measures, novelty, cognitive load, user control, elaboration, process evaluation questions, and podcast and Twitter usage.	Intention-to-treat analyses showed weight loss did not differ by group at 6 months. Days/weeks of reported diet mointoring did not differ between Podcast+Mobile and Podcast groups.
Brindal et al. (2012)	n= 8112<br>Retention Rate= 5.2%<br>Mean Age= 45.0 (11.9)<br>83% female<br>Mean BMI= 34.0 (6.5)<br>Healthy adults with BMI >25	Australia	Supportive website: Information-based site with interactive tools such as real-time dietary compliance visulizations, an interactive meal planner, and a social networking platform including personal profiles, friend networks, blogs, discussion forums, and news feeds.<br>Personalized- supportive website: Identical to supportive version with a personalized meal planner.	Information-based: Static non-interactive version of the weight loss program with no social networking platform.	Social networking platform including personal profiles, friend networks, blogs, discussion forums, and news feeds	Primary: Body weight at baseline and 12 weeks. Secondary: Usage of website, attitudes toward website.	Intention-to-treat analyses using multiple imputations revealed that there were no statistically significant differences in weight loss between sites. Within supportive conditions, the level of use of the online weight tracker was predictive of weight loss. Attrition was high for all three websites.
Napolitano et al. (2013)	n= 52<br>Retention Rate= 96%<br>Mean Age= 20.5 (2.2)<br>86.5% female<br>Mean BMI= 31.4 (5.3)<br>Healthy college students age 18-29 with BMI 25-50	US	Facebook: Private Facebook group as a portal to access handouts, podcasts, event invitations, and intervention content via group postings and Facebook mail.<br>Facebook Plus: Separate private Facebook group plus intervention targets and feedback via text messaging (weight goals, tips on self-monitoring), as well as a digital scale, pedometer, Calorie King book, and measuring utensils.	Waitlist control.	Facebook	Primary: BMI at weeks 4 and 8. Secondary: Physical activity behavior, goal setting and planning, physical activity self-efficacy, weight self-efficacy, adapted social support for diet and exercise, engagement or compliance, consumer satisfaction.	Using ANCOVA, Facebook Plus group had significantly greater weight loss than Facebook and Waiting List at 8 weeks.
**WEIGHT MAINTENANCE**							
Harvey-Berino et al. 2004	n= 255Retention Rate=69%Mean Age= 45.8 (8.9)82% femaleMean BMI= 31.8 (4.1)Adults age 18 and older with BMI≥25	US	All participants completed a 6 month weight loss intervention conducted over interactive television. They were then randomized into 1 of 3 groups: 1. internet support (IS), 2. frequent in-person support (F-IPS) or 3. minimal	Please see interventions. No other control group.	Chatroom and bulletin board in IS group	Primary: Body weight, height, energy intake, and energy expended were taken at 0, 6, 12, and 18 months.<br>Secondary: Computer	Intention-to-treat analysis showed no significant differences among the groups in weight loss from baseline to 18 months.

			in-person support (M-IPS), for 12 months of weight loss maintenance. F-IPS (bi-weekly ITV and group therapist session) and IS (website with chatroom and bulletin board) differed only in method of delivery of the multi-component weight maintenance program. M-IPS had 6 months of monthly in-person (ITV) therapy and no contact for last 6 months.			experience and comfort, attendance at meetings and chat sessions, adherence to monitoring, social influence components.	
Cussler et al. 2008	n= 161<br>Retention Rate=69%<br>Mean Age= 48 (4.4)<br>100% female<br>Mean BMI= 31.1 (3.8)<br>Healthy, non-smoking adults age 40- 55 with BMI 25 -38	US	All participants completed 4 month weight loss intervention. Intervention group had access to an internet group hosting communication tools (private mail, group mail, message board, and chat room), progress monitoring tools, and dietary/physical activity information. Participants were required to enter weight, diet, and exercise on a regular basis.	Control participants had no further contact with study staff after 4 month weight loss intervention except for testing. They were also permitted to meet and practice the principles learned during the 4 -month program.	Bulletin board and chatrooms in interventio n group	Primary: BMI, body fat percentage, and total body fat mass were measured at 0, 4, and 16 months. Secondary: Physical activity and dietary assessment were measured at 0, 4, and 16 months.	Both groups had no difference in the amount of weight regained at 16 months. In within-group analyses, Internet diet log entries correlated with weight change and moderately with change in exercise energy expenditure.
