# Supplementary material for: The Role of Social Media in Online Weight Management: Systematic Review
Source: J Med Internet Res. 2013 Nov 28;15(11):e262. doi: 10.2196/jmir.2852 (PMC3868982; doi:10.2196/jmir.2852)
Supplement: Supplementary file 3 [file jmir_v15i11e262_app3.pdf]

Appendix C. Jadad scale to evaluate risk for bias

	Randomized	Double-blinded	Description of withdrawals & dropouts	Method for randomization	Method for Blinding	Jadad Score (Max =5)	Risk of Bias
**Diet**							
Verheijden (2004)	1	0	1	1	1	4	Low
**Physical Activity**							
Hurling (2007)	1	0	1	0	0	2	Moderate
Ferney (2009)	1	0	0	1	0	2	Moderate
Liebreich (2009)	1	0	0	0	1	1	High
Richardson (2010)	1	0	1	1	0	3	Moderate
Cavallo (2012)	1	0	0	0	0	1	High
**Diet and Physical Activity**							
Tate (2001)	1	0	1	0	0	2	Moderate
Tate (2003)	1	0	1	1	0	3	Moderate
Womble (2004)	1	0	1	0	0	2	Moderate
Tate (2006)	1	0	0	1	0	2	Moderate
Gold (2007)	1	0	1	0	0	2	Moderate
Webber (2007)	1	0	0	0	0	1	High
Morgan (2009)	1	1	1	1	1	5	Low
Sternfeld (2009)	1	0	0	0	0	1	High
Harvey-Berino (2010)	1	0	1	1	0	3	Moderate
Turner McGrievy (2011)	1	0	0	1	0	2	Moderate
Brindal (2012)	1	1	1	1	1	5	Low
Napolitano (2013)	1	0	0	0	0	1	High
**Weight Maintenance**							
Harvey-Berino (2004)	1	0	1	0	0	2	Moderate
Cussler (2008)	1	0	0	0	0	1	High
					Median Score	2	Moderate

Score of 0-1= High Risk of Bias

Score of 2-3= Moderate Risk of Bias

Score of 4-5= Low Risk of Bias
