# Supplementary material for: The Role of Social Media in Online Weight Management: Systematic Review
Source: J Med Internet Res. 2013 Nov 28;15(11):e262. doi: 10.2196/jmir.2852 (PMC3868982; doi:10.2196/jmir.2852)
Supplement: Supplementary file 4 [file jmir_v15i11e262_app4.pdf]

Appendix D. Randomized control trial quality scores adapted from Norman et al.

	Individual randomization	Control group	Social Media Isolated	Pre-post test design	Retention 80%	Baseline groups equivalent	Missing data	Sample size calculation	Validated measures	Score (Max= 9)	Quality
**Diet**											
Verheijden (2004)	Y	Y	N	Y	Y	Y	Y	Y	Y	8	High
**Physical Activity**											
Hurling (2007)	Y	Y	N	Y	Y	Y	Y	N	Y	7	High
Ferney (2009)	Y	Y	N	Y	Y	N	Y	N	Y	6	Moderate
Liebreich (2009)	Y	Y	N	Y	Y	N	Y	N	Y	6	Moderate
Richardson (2010)	Y	Y	Y	Y	N	N	Y	Y	Y	7	High
Cavallo (2012)	Y	Y	N	Y	Y	Y	N	Y	Y	8	High
**Diet and Physical Activity**											
Tate (2001)	Y	Y	N	Y	N	Y	N	Y	Y	6	Moderate
Tate (2003)	Y	Y	N	Y	Y	Y	Y	N	Y	7	High
Womble (2004)	Y	Y	N	Y	N	Y	Y	Y	Y	7	High
Tate (2006)	Y	Y	N	Y	Y	Y	Y	Y	Y	8	High
Gold (2007)	Y	Y	N	Y	N	Y	Y	N	Y	6	Moderate
Webber (2007)	Y	Y	N	Y	Y	Y	Y	Y	Y	8	High
Morgan (2009)	Y	Y	N	Y	Y	Y	Y	Y	Y	8	High
Sternfeld (2009)	Y	Y	N	Y	N	N	Y	N	Y	5	Moderate
Harvey-Berino (2010)	Y	Y	N	Y	Y	Y	Y	Y	Y	8	High
Turner McGrievy (2011)	Y	Y	N	Y	Y	Y	Y	Y	Y	8	High
Brindal (2012)	Y	Y	N	Y	N	U	Y	N	Y	5	Moderate
Napolitano (2013)	Y	Y	N	Y	Y	Y	Y	N	Y	7	High
**Weight Maintenance**											
Harvey-Berino (2004)	Y	Y	N	Y	N	Y	Y	Y	Y	7	High
Cussler (2008)	Y	Y	N	Y	N	Y	Y	N	Y	6	Moderate
									Median Score	7.0	High

Scores of 7-9= High Quality

Scores of 4-6= Moderate Quality

Scores of 0-3= Low Quality
